# Plasma inflammatory biomarker profiles across the Alzheimer's disease spectrum in the Bio‐Hermes cohort

**DOI:** 10.1002/alz.71257

**Published:** 2026-03-12

**Authors:** Zain Hussain, Dominic Ng, Samuel Leighton, Fani Deligianni, Craig Ritchie, Jonathan Cavanagh

**Affiliations:** ^1^ College of Medical, Veterinary and Life Sciences University of Glasgow Glasgow Scotland UK; ^2^ Scottish Brain Sciences Edinburgh Scotland UK; ^3^ Centre for Clinical Brain Sciences University of Edinburgh Edinburgh Scotland UK; ^4^ School of Computing Science University of Glasgow Glasgow Scotland UK; ^5^ School of Medicine University of St Andrews St Andrews Scotland UK

**Keywords:** Alzheimer's disease, amyloid, *APOE* ε4, biomarkers, cytokines, inflammation

## Abstract

**INTRODUCTION:**

Inflammation contributes to Alzheimer's disease (AD), but its stage‐specific and amyloid‐dependent patterns remain unclear.

**METHODS:**

We analyzed 964 participants from the Bio‐Hermes cohort (cognitively normal [CN] = 404, mild cognitive impairment [MCI] = 302, mild AD = 258). Plasma levels of 32 cytokines, neurofilament light chain (NfL) and glial fibrillary acidic protein (GFAP) were quantified alongside core AD biomarkers. Associations with cognition, amyloid, apolipoprotein E (*APOE*) ε4, and clinical outcomes were assessed using analysis of covariance, partial correlations, and regression models.

**RESULTS:**

Twenty‐four cytokines, NfL, and GFAP differed across cognitive groups. Amyloid stratification revealed a core amyloid‐independent profile (14 cytokines + NfL) and a broader amyloid‐specific profile including GFAP, interleukin (IL)‐1β, and IL‐18, implicating microglial inflammasome and astrocytic activation. Stage‐dependent patterns suggested inflammation may act as early driver, concurrent process, or late amplifier. Paradoxical associations (e.g., eotaxin‐2, IL‐2R with better memory) and *APOE* ε4‐linked immune differences indicated context‐dependent roles.

**DISCUSSION:**

This exploratory study reveals biologically plausible, inflammatory heterogeneity in AD and highlights plasma cytokine profiles as candidate biomarkers and therapeutic targets, warranting investigation.

## Highlights

- The stage‐ and amyloid‐specific patterns of systemic inflammation in AD remain unclear.
- We conducted an exploratory analysis of 32 plasma cytokines, NfL, and GFAP in 964 Bio‐Hermes participants.
- Distinct amyloid‐independent and amyloid‐specific inflammatory signatures were identified, and the amyloid‐specific profile was characterized by IL‐1β, IL‐18, and GFAP, implicating inflammasome‐linked glial activation.
- Certain cytokines showed paradoxical associations with better cognition, and *APOE* ε4 shaped immune profiles.
- Findings suggest biologically distinct inflammatory subtypes of AD, highlighting potential biomarkers and targeted immunomodulatory targets.

## 1 BACKGROUND

Alzheimer's disease (AD), the most common cause of dementia, is a progressive neurodegenerative disorder characterized by cognitive decline and the accumulation of amyloid beta (Aβ) plaques and neurofibrillary tangles in the brain^1^ ^,^.^2^, ^3^ The role of amyloid in AD is well established, and the recent regulatory approval of anti‐amyloid monoclonal antibody therapies represents an important therapeutic milestone. However, their effects are clinically modest and do not reverse cognitive deficits.^4^ This limited benefit may reflect that treatment is initiated too late, that other processes independently drive disease progression, or that benefits are restricted to specific subgroups.^5^, ^6^

Beyond anti‐amyloid therapies, several inflammatory pathways have been evaluated as therapeutic targets in AD, including interleukin (IL)‐1β blockade, tumor necrosis factor alpha (TNF‐α) inhibitors, NOD‐like receptor protein 3 (NLRP3)‐inflammasome inhibitors, and IL‐17A pathway modulation.^7^, ^8^, ^9^ These reflect a growing interest in immune‐modifying strategies; however, mixed clinical results highlight the need to better characterize inflammatory heterogeneity. Collectively, these factors highlight the multifaceted nature of AD, where cognitive decline arises from a complex interplay of pathologies beyond amyloid accumulation, underscoring the need to explore other mechanisms of disease.

Neuroinflammation is increasingly recognized as a central element of AD pathogenesis. Traditionally viewed as a reactive consequence of neuronal damage,^10^ growing evidence suggests that inflammation actively contributes to disease onset and progression through sustained glial activation.^11^ Central to this process are microglia and astrocytes, which transition to reactive states and release distinct inflammatory mediators. These pathological changes can be quantified via fluid biomarkers, including glial fibrillary acidic protein (GFAP) (reflecting astrogliosis), soluble triggering receptor expressed on myeloid cells 2 (indicating microglial activation), and signaling cytokines (e.g., TNF‐α, IL‐1β).^12^ While cerebrospinal fluid (CSF) offers higher diagnostic specificity for central neuroinflammation, its invasiveness limits widespread use. Plasma levels reflect a composite of central and peripheral immune activity,^13^ providing an important window into the systemic inflammatory context of AD, and offer a scalable, non‐invasive alternative for population‐level screening and interventions. This systemic perspective is particularly relevant given that inflammation is associated with multiple AD risk factors, including age, sex, infection, cardiovascular disease, diabetes, smoking, and depression, yet its exact role in the neurobiology of AD remains unclear. An important question remains whether inflammation is a driver, concurrent process, or consequence of neurodegeneration.

Previous studies investigating inflammation in AD yielded mixed findings, with some reporting elevated pro‐inflammatory cytokines in symptomatic stages, while others observe compensatory or anti‐inflammatory responses. Such inconsistencies likely reflect differences in study design, analytical sensitivity, or population characteristics, underscoring the complexity of immune involvement in AD. For example, a large United Kingdom Alzheimer's Disease Research Center (UK‐ADRC) study examined associations between six AD plasma biomarkers (Aβ40, Aβ42, Aβ42/40, tau phosphorylated at threonine 181 [p‐tau181], total tau [t‐tau], and neurofilament light chain [NfL]) and five inflammatory biomarkers (TNFα, IL6, IL8, IL10, and GFAP).^14^ They reported that increasing AD pathology corresponded to higher systemic inflammatory marker levels, with the strength and direction of these associations varying across cognitive stages, suggesting a dynamic and stage‐dependent relationship between inflammation and neurodegeneration.

Despite growing evidence, the specific cytokine signatures underlying amyloid‐dependent versus amyloid‐independent processes remain poorly characterized. The large Bio‐Hermes cohort offers a unique opportunity in this context, incorporating 65 plasma cytokines, eight neurodegeneration‐related plasma biomarkers, and detailed clinical and demographic data (e.g., apolipoprotein E [*APOE*] status, amyloid status).^15^ This enables examination of how systemic inflammatory markers relate to key AD risk factors (cognition, amyloid, and *APOE* ε4 status) and interact with established AD biomarkers. A clearer understanding of these inflammatory profiles may offer insight into the biological pathways underlying AD and inform the development of diagnostic, prognostic, or therapeutic tools.

This was an exploratory, hypothesis‐generating study, which sought to:

1. Characterize baseline associations between plasma inflammatory markers, neuronal damage markers (NfL, GFAP), and key clinical/biological factors (cognitive status, amyloid status, *APOE* ε4 carrier status).
2. Examine the correlations between cognition‐associated inflammatory markers and core AD biomarkers and assess how relationships differ by cognitive status.
3. Assess the associations between cognition‐associated inflammatory markers and clinical outcome measures related to cognition (MMSE), functional ability (FAQ), memory (RAVLT), and depressive symptoms (GDS).

## 2 METHODS

### 2.1 Study participants and ethical approval

We analyzed data and biospecimens from the Bio‐Hermes study (*N* = 1001), a cross‐sectional initiative designed to evaluate biomarkers for AD. The recruitment strategies from US clinical trial sites, detailed participant eligibility criteria, and overarching study design have been described previously.^15^ Of relevance to our analysis is the exclusion criteria, which minimize inflammatory confounding, whereby those with a history of significant central nervous system (CNS) disorder other than AD, as well as those with active systemic inflammatory conditions or current use of immunosuppressive medications, were excluded. All protocols were approved by a central Institutional Review Board (Advarra, Protocol ID: Pro00046018), and all participants or their legally authorized representatives provided written informed consent.

The current analyses focus on a subset of 964 participants aged 60 to 85 with complete data, who were categorized as cognitively normal (CN; *n* = 404), mild cognitive impairment (MCI; *n* = 302), or having mild AD dementia (mild AD; *n* = 258) based on using a National Institute on Aging–Alzheimer's Association (NIA‐AA)‐based diagnostic framework with study‐specified cognitive and functional thresholds (Mini‐Mental State Examination [MMSE], and Rey Auditory Verbal Learning Test [RAVLT] delayed recall, Functional Activities Questionnaire [FAQ]), as detailed in the primary study protocol.^15^

**RESEARCH IN CONTEXT**

1. **Systematic review**: We reviewed the published literature on plasma biomarkers of AD and inflammation using PubMed and major meeting abstracts. Previous studies reported links between individual cytokines and AD pathology, but comprehensive analyses spanning multiple cytokines in large, deeply phenotyped cohorts remain scarce.
2. **Interpretation**: Using the Bio‐Hermes cohort, we identified distinct amyloid‐independent and amyloid‐specific inflammatory signatures, stage‐dependent associations with cognition, and paradoxical links suggesting compensatory or context‐dependent immune responses. These findings extend current understanding by highlighting systemic inflammatory heterogeneity in AD and its potential mechanistic and clinical relevance.
3. **Future directions**: Longitudinal validation is needed to test whether inflammatory signatures predict progression, to integrate plasma with CSF and imaging measures, and to evaluate composite cytokine risk scores. Trials of pathway‐specific immunomodulation (e.g., targeting NLRP3, IL‐17A, or TNF signaling) should consider stratification by inflammatory subtype to enhance precision and therapeutic efficacy.

### 2.2 Clinical assessments and biomarker quantification

As described in the parent study,^15^ key clinical and cognitive assessments used for cohort characterization or as outcome measures included the MMSE, RAVLT (delayed recall), FAQ, and the Geriatric Depression Scale (GDS).

Peripheral blood samples were collected at the initial screening visit and processed according to the standardized Bio‐Hermes protocol. *APOE* genotyping was performed by C2N Diagnostics (St. Louis, MO, USA) to determine ε4 carrier status (carrier or non‐carrier).

A panel of plasma biomarkers was quantified. Sixty‐five inflammatory cytokines were measured from plasma samples using the ProcartaPlex Human Immune Monitoring panel (Thermo Fisher Scientific). Plasma NfL and GFAP concentrations were measured via Single Molecule Array (Simoa) at Quanterix Laboratories. Core AD biomarkers were quantified using distinct platforms: plasma Aβ40, Aβ42, and Aβ42/Aβ40 ratios were measured via liquid chromatography‐tandem mass spectrometry (LC‐MS/MS) (C2N Diagnostics), while p‐tau181, p‐tau217, and t‐tau were quantified using the Simoa HD‐X platform (Quanterix).

Brain amyloid positron emission tomography (PET) scans were required to be completed within 60 days of when the informed consent was signed and a lumbar puncture within 30 days of the coagulation panel for sites without PET access. Brain amyloid status (positive/negative) was determined primarily by Florbetapir (18F) PET (positivity defined as Centiloid > 24.1). In a small minority of cases (<4%) where PET was unavailable due to site‐specific constraints, status was determined by CSF Aβ42/Aβ40 ratio (positivity defined as ratio < 0.068), as per the primary study protocol.^15^

### 2.3 Statistical analysis

Of the 65 cytokines measured, 33 were excluded because >50% of values were undetectable. For the remaining 32 cytokines, undetectable values (coded as < out of range) were imputed, using a conservative substitution method, with half of the lowest observed non‐zero concentration for that analyte.^16^ The proportion of undetectable values for each cytokine is provided in Table S1. Following visual inspection of variable distributions, all retained cytokines, as well as plasma NfL and GFAP, were log‐transformed, consistent with standard analytical practice in cytokine quantification studies. Plasma AD biomarkers (Aβ40, Aβ42, Aβ42/40, p‐tau181, p‐tau217, t‐tau) distributions did not require transformation and were analyzed on their original scale, an approach aligned with prior Bio‐Hermes analyses.^15^ Pairwise Pearson correlation analyses were conducted across all log‐transformed inflammatory biomarker pairs to characterize inter‐biomarker relationships and correlation coefficients (*r*) used to visualize co‐expression patterns.

Analysis of covariance (ANCOVA) was used to determine mean differences in log‐transformed biomarker levels across three key factors: (1) cognitive status (CN, MCI, mild AD), (2) *APOE* ε4 status (carrier vs non‐carrier), and (3) amyloid status (positive vs negative). The ANCOVA comparing cognitive groups was also performed stratified by amyloid status (positive vs negative). All ANCOVA models were adjusted for age and sex. Tukey's Honestly Significant Difference (HSD) test was employed for post hoc pairwise comparisons following ANCOVAs on cognitive status. To account for multiple comparisons, the Benjamini‐Hochberg false discovery rate (FDR) procedure was applied, with findings considered statistically significant if the *q* value was less than 0.05.

Inflammatory markers identified as associated with cognitive status in the primary ANCVOA (FDR‐corrected; 24 cytokines plus NfL, GFAP) were carried forward for all secondary inferential analyses. Partial Pearson correlations, adjusting for age, sex, and *APOE* ε4 status, were used to examine relationships between this subset of inflammatory markers and core AD biomarkers, within each cognitive group. To assess associations between these inflammatory biomarkers and clinical outcome scores (MMSE, FAQ, RAVLT, GDS), linear regression models were employed, adjusting for age, sex, education, and *APOE* ε4 status, both across the cohort and stratified by cognitive status. Given that these secondary analyses were restricted to a subset of significant markers, nominal *p* values (uncorrected) were used. For completeness, correlations between all 32 retained cytokines and AD biomarkers are additionally provided in the Supplementary Material (Table S2).

Statistical analyses and data visualization were performed using Python, version 3.10, within the Alzheimer's Disease Data Initiative (ADDI) Jupyter Notebook environment.

## 3 RESULTS

### 3.1 Study participant demographics

The baseline characteristics of 964 participants from the Bio‐Hermes study are detailed in Table 1, including demographic information, cognitive measure scores, and plasma biomarkers levels across the cognitive groups (CN, MCI, and mild AD).

**TABLE 1:** Baseline demographic, clinical, and biomarker characteristics of study participants by cognitive group.

	Cognitive normal (*n* = 404)	Mild cognitive impairment (*n* = 302)	Mild Alzheimer's disease (*n* = 258)	Total population (964)
Age, mean (SD)	70.38 (6.44)	72.27 (6.82)	74.44 (6.02)	72.07 (6.66)
Female/Male (*n*/*n*)	155 (38.4%)/249 (61.6%)	140 (46.4%)/162 (53.6%)	125 (48.4%)/133 (51.6%)	420 (43.5%)/544 (56.4%)
Education, years, mean (SD)	15.78 (2.47)	15.50 (2.77)	14.76 (3.04)	15.42 (2.75)
Amyloid positive/negative (*n*,*n*)	83 (20.5%)/304 (75.2%)	100 (33.1%)/188 (62.3%)	96 (37.2%)/150 (58.1%)	333 (34.5%)/588 (60.9%)
ApoE ε4 carrier/non‐carrier, *n*/*n*	135 (33.4%)/269 (66.6%)	114 (37.7%)/188 (62.3%)	113 (43.8%)/145 (56.2%)	365 (37.8%)/600 (62.2%)
Ethnicity				
Non‐Hispanic White, *n*	320 (79.2%)	227 (75.2%)	168 (65.1%)	716
Hispanic, *n*	40 (9.9%)	34 (11.3%)	49 (19.0%)	111
Non‐Hispanic Black, *n*	28 (6.9%)	31 (10.3%)	31 (12.0%)	102
Other, *n*	16 (4.0%)	10 (3.3%)	10 (3.9%)	36
Cognitive measures				
MMSE_Total, mean (SD)	28.42 (1.47)	27.14 (2.00)	23.15 (2.55)	26.61 (2.92)
RAVLT T score, mean (SD)	47.49 (13.45)	37.30 (11.73)	31.08 (12.26)	39.90 (14.35)
FAQ total, mean (SD)	0.77 (1.66)	3.59 (4.54)	9.46 (6.87)	3.98 (5.71)
GDS, mean (SD)	1.46 (1.60)	2.26 (2.13)	2.20 (1.87)	1.91 (1.89)
AD plasma biomarkers				
Aβ40, mean (SD)	109.87 (37.44)	112.27 (31.00)	104.53 (44.75)	109.19 (37.81)
Aβ42, mean (SD)	5.78 (1.83)	5.79 (1.74)	5.08 (2.26)	5.59 (1.95)
Aβ42/40, mean (SD)	0.05 (0.01)	0.05 (0.01)	0.06 (0.12)	0.06 (0.06)
*p*‐tau181, pg/mL, mean (SD)	16.96 (10.61)	19.42 (11.28)	23.34 (16.74)	19.45 (12.97)
*p*‐tau217, pg/mL, mean (SD)	2.36 (1.31)	3.16 (2.50)	4.04 (2.62)	3.06 (2.23)
*t*‐tau pg/mL, mean (SD)	2.17 (1.98)	2.46 (5.36)	2.27 (4.38)	2.29 (3.97)
GFAP, mean (SD)	149.02 (79.32)	185.89 (165.40)	224.00 (116.71)	180.76 (125.42)
NfL, mean (SD)	24.34 (12.67)	32.51 (29.46)	41.65 (29.30)	31.56 (24.84)

*Note*: Baseline demographic, clinical, and biomarker data are presented as mean (SD) or n (%).

Abbreviations: Aβ, amyloid beta; AD, Alzheimer's disease; CN, cognitively normal; FAQ, Functional Activities Questionnaire; GDS, Geriatric Depression Scale; GFAP, glial fibrillary acidic protein; MCI, mild cognitive impairment; MMSE, Mini‐Mental State Examination; NfL, neurofilament light chain; p‐tau, phosphorylated tau; RAVLT, Rey Auditory Verbal Learning Test; SD, standard deviation; t‐tau, total tau.

Other abbreviations are defined at first use in the text.

The most prevalent comorbidities that were identified were hypertension in 42% of participants, hyperlipidemia in 25%, a history of depression in 18%, hypercholesterolemia in 17%, anxiety in 17%, hypothyroidism in 12%, and insomnia in 12%. Commonly reported medication use from highest to lowest was as follows: acetylsalicylic acid (25.0%), atorvastatin (23.3%), colecalciferol (vitamin D3) (16.0%), levothyroxine (14.9%), amlodipine (14.6%), and donepezil (10%).

### 3.2 Inter‐relationships among plasma inflammatory biomarkers

**FIGURE 1:**
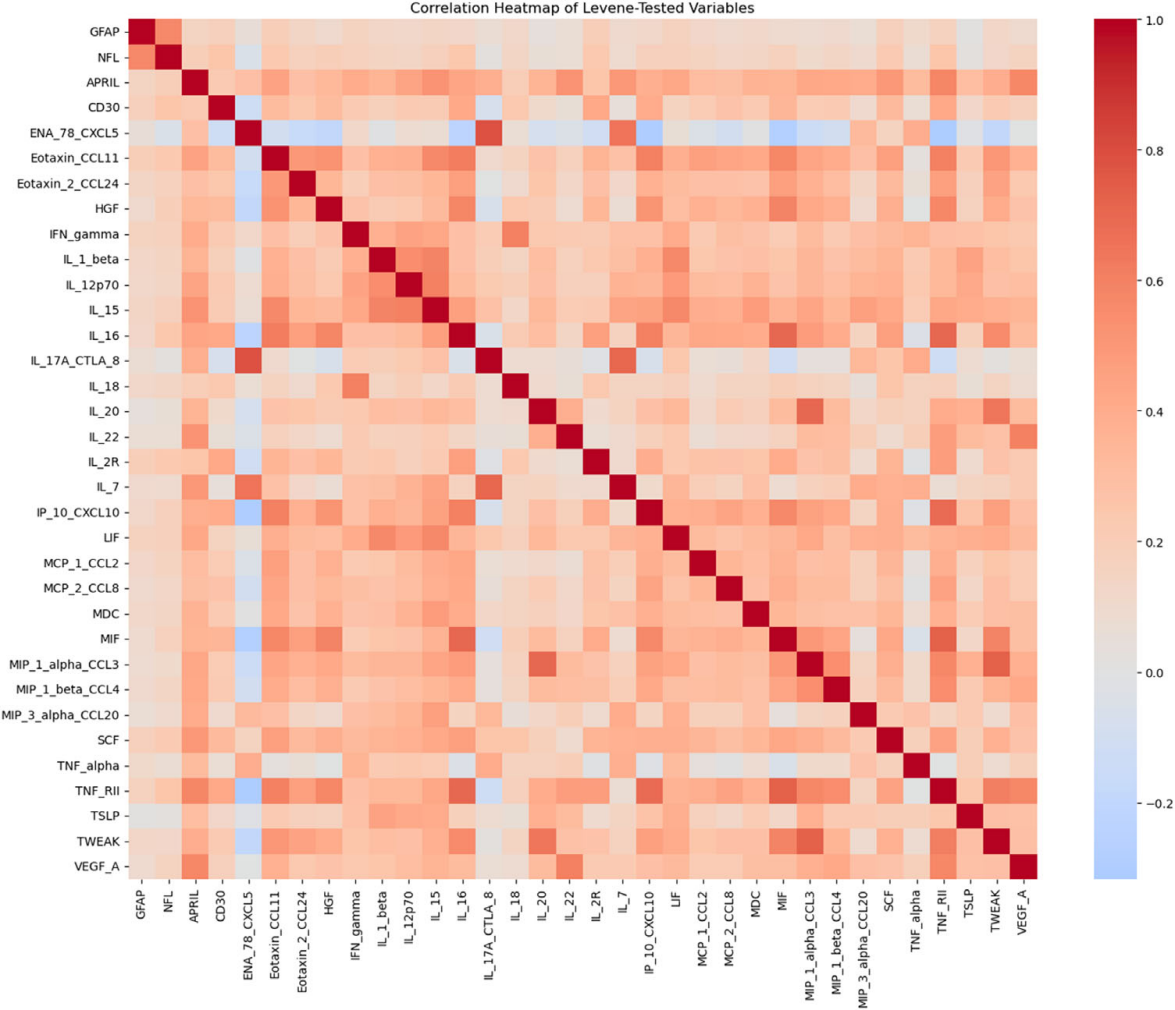
Correlation heatmap of plasma inflammatory biomarkers. Heatmap showing pairwise Pearson's correlation coefficients (*r*) among plasma cytokines (log‐transformed) and neurodegeneration biomarkers (neurofilament light chain and glial fibrillary acidic protein). Red indicates stronger positive correlations, while blue negative correlations. The diagonal represents self‐correlations (*r* = 1).

We first examined correlations among the 34 measurable inflammatory markers. Distinct co‐regulated groups were identified based on strong pairwise correlations (*r* ≥ 0.5) observed on the heatmap (Figure 1), including a large “hub” centered on TNF‐RII (positively associated with MIF, IL‐16, IP‐10, TWEAK, eotaxin [CCL11], APRIL, HGF, VEGF‐A, and MIP‐1α/β). A second “hub” was defined by the IL‐17A axis, with high correlations to ENA‐78 (CXCL5) and IL‐7. Additional expected partnerships were observed, such as the interferon‐γ (IFN‐γ)/IL‐18 pair, IL‐1β with IL‐15 and LIF, and the positive link between neurodegeneration markers GFAP and NfL. These coordinated patterns suggest that systemic inflammation in AD reflects activation of specific biological pathways rather than non‐specific immune activation, which informed interpretation of subsequent analysis.

### 3.3 Inflammatory biomarker levels by clinical and pathological groupings

We used age‐ and sex‐adjusted ANCOVA models to compare the levels of 34 plasma inflammatory biomarkers across key clinical and biological groups. Findings were statistically significant after correcting for multiple comparisons (FDR *q* < 0.05).

#### 3.3.1 Differences across cognitive groups

We found 24 of 32 cytokines, along with NfL and GFAP, differed significantly across CN, MCI, and mild AD groups.

Notably, significant stepwise increases from CN through MCI to AD were observed for NfL, GFAP, and chemokine eotaxin‐2 (CCL24), indicating a response tracking with disease severity. Further, cytokines were found to be elevated in the mild AD group only compared to both CN and MCI, indicating a broader activation of multiple inflammatory pathways at this stage. These markers included those from distinct groupings identified in our correlation analysis, including cytokines from the TNF‐RII hub (TNF‐RII, IL‐16, MIF, TWEAK, IP‐10, HGF, APRIL), the innate immune pathway (IL‐1β, IL‐15, LIF), and the IL‐17A axis (IL‐17A, IL‐7). Other cytokines in this pattern included eotaxin (CCL11), MCP‐1 (CCL2), MDC, MIP‐1β (CCL4), SCF, and TNF‐α.

**FIGURE 2:**
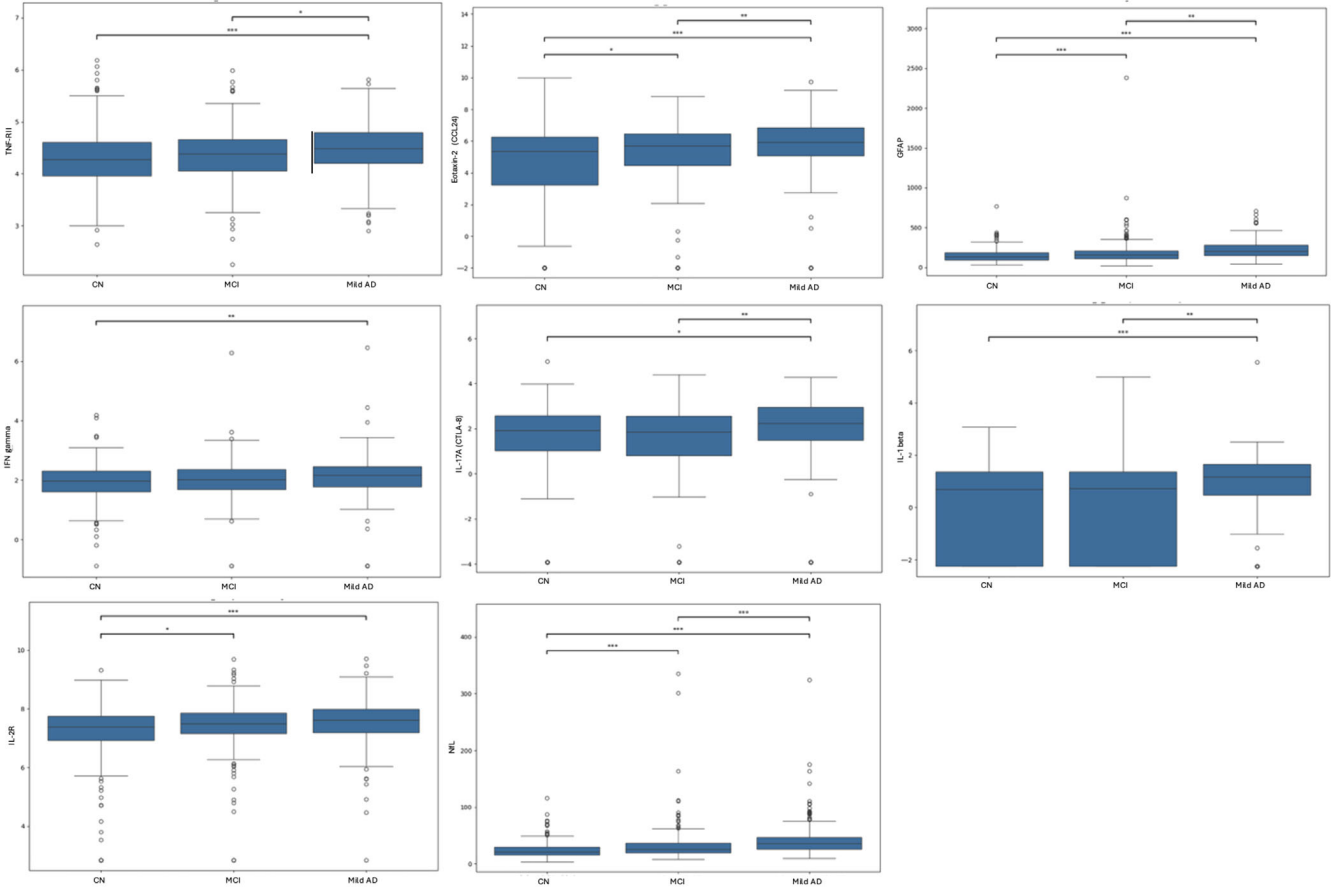
Boxplots of plasma inflammatory and neurodegenerative biomarkers across cognitive groups. Boxplots display neurofilament light chain, glial fibrillary acidic protein, and selected log‐transformed plasma levels of cytokines across cognitively normal, mild cognitive impairment, and mild Alzheimer's disease participants. Error bars represent interquartile ranges, with horizontal lines indicating medians and open circles denoting outliers. Statistical significance is represented as follows: *p < 0.05, **p < 0.01, ***p < 0.001.

Notably, IL‐2R, a marker of T‐cell activation, was significantly elevated in both MCI and mild AD groups compared to the CN group but did not differ between MCI and mild AD groups. Another distinct pattern was observed for four cytokines (IFN‐γ, IL‐18, MCP‐2/CCL8, and MIP‐1α/CCL3) that were significantly higher in the mild AD group only compared to the CN group, possibly suggesting a gradual increase in levels. See Figure 2 for boxplots of selected cytokines from this analysis and Table 2 for a summary of patterns of cytokine changes across cognitive groups.

**TABLE 2 alz71257-tbl-0002:** Summary of stage‐dependent patterns of plasma inflammatory markers across cognitive groups.

Pattern of change	Cluster/pathway	Cytokines
Stepwise increase CN → MCI → AD	Neurodegeneration/astrocytic	NfL, GFAP
	Chemokine	Eotaxin‐2 (CCL24)
Elevated only in mild AD versus CN and MCI	TNF‐RII hub	TNF‐RII, IL‐16, MIF, TWEAK, IP‐10 (CXCL10), HGF, APRIL
	Innate immune	IL‐1β, IL‐15, LIF
	IL‐17A axis	IL‐17A (CTLA‐8), IL‐7
	Other cytokines	Eotaxin (CCL11), MCP‐1 (CCL2), MDC, MIP‐1β (CCL4), SCF, TNF‐α
Elevated in both MCI and mild AD versus CN	T‐cell activation	IL‐2R
	Mixed	IFN‐γ, IL‐18, MCP‐2 (CCL8), MIP‐1α (CCL3)

*Note*: Cytokines are grouped by biological hub or pathway, with patterns defined as (i) stepwise increase across CN → MCI → mild AD, (ii) elevation restricted to mild AD, or (iii) elevation in both MCI and mild AD compared with CN.

Abbreviations: AD, Alzheimer's disease; APRIL, a proliferation‐inducing ligand; CCL, C–C motif chemokine ligand; CN, cognitively normal; GFAP, glial fibrillary acidic protein; HGF, hepatocyte growth factor; IFN, interferon; IL, interleukin; LIF, leukemia inhibitory factor; MCI, mild cognitive impairment; MCP, monocyte chemoattractant protein; MDC, macrophage‐derived chemokine; MIF, macrophage migration inhibitory factor; MIP, macrophage inflammatory protein; NfL, neurofilament light chain; SCF, stem cell factor; TNF, tumor necrosis factor; TWEAK, TNF‐like weak inducer of apoptosis.

#### 3.3.2 Amyloid status modulates inflammatory differences across cognitive groups

We carried out a stratified analysis, repeating the comparison of biomarker levels across cognitive groups separately for amyloid‐positive and amyloid‐negative participants (Table S3).

A substantial component of the inflammatory response appeared to be independent of amyloid pathology, with 14 cytokines and NfL found to be significantly associated with cognitive status in both amyloid‐positive and amyloid‐negative subgroups. These included APRIL, eotaxin‐2 (CCL24), eotaxin (CCL11), HGF, IL‐16, IL‐7, IP‐10 (CXCL10), LIF, MCP‐2 (CCL8), MDC, MIF, MIP‐1β (CCL4), SCF, and TWEAK. IL‐2R showed an association with cognitive status only within the amyloid‐negative group.

Seven cytokines and GFAP had an association with cognitive status only within the amyloid‐positive subgroup. These included cytokines from the innate immune hub (IL‐1β, IL‐15), as well as IL‐18, IL‐12p70, MCP‐1 (CCL2), MIP‐1α (CCL3), and TNF‐RII.

We also compared plasma inflammatory biomarkers between amyloid‐positive and amyloid‐negative participants across the cohort. Amyloid‐positive individuals showed significantly higher levels of GFAP and NfL (*q* < 0.001) compared to amyloid‐negative individuals. No other inflammatory cytokines showed a significant difference between the two groups in this main analysis.

#### 3.3.3 Association with *APOE* ε4 carrier status

We found *APOE* ε4 carrier status to be associated with a distinct inflammatory profile. Carriers of *APOE* ε4 had significantly higher levels of GFAP (*q* = 0.004) and lower levels of IL‐15, IL‐20, and MCP‐2/CCL8 (all *q* = 0.038) compared to non‐carriers.

### 3.4 Correlations between inflammatory biomarkers and AD biomarkers

**FIGURE 3:**
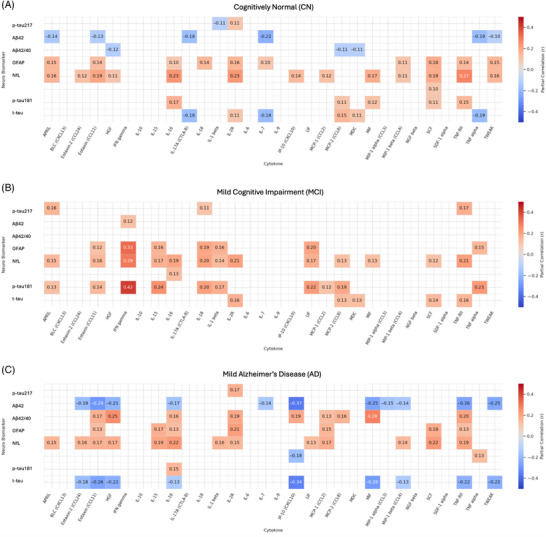
Partial correlations between plasma cytokines and neurodegeneration biomarkers across cognitive groups. Heatmaps show partial Pearson correlations (*r*) between plasma cytokines (log‐transformed) and neurodegenerative biomarkers were computed separately for each cognitive group: (A) cognitively normal, (B) mild cognitive impairment, and (C) mild Alzheimer's disease. All analyses were adjusted for age, sex, and apolipoprotein E ε4 carrier status. Only statistically significant correlations (*p* < 0.05, uncorrected) are displayed. Color intensity reflects correlation strength, with red indicating positive and blue indicating negative associations.

Partial correlation analyses, adjusted for age, sex, and *APOE* ε4 status, were conducted within each clinical group to examine associations between the 24 significant cognition‐associated inflammatory biomarkers identified in the primary analysis and core AD biomarkers (Figure 3 shows correlation matrices for CN, MCI, and mild AD groups).

In the CN group, modest positive correlations were observed between NfL and several cytokines, including TNF‐RII (*r* = 0.27, *p* < 0.001), CD30 (*r* = 0.24, *p* < 0.001), IL‐16 (*r* = 0.23, *p* < 0.001),[Table alz71257-tbl-0002] IL‐2R (*r* = 0.23, *p* < 0.001), and eotaxin (CCL11) (*r* = 0.19, *p* < 0.001). GFAP correlated positively with SCF (*r* = 0.18, *p* < 0.001).

In the MCI group, stronger correlations were observed between inflammatory biomarkers and tau pathology. IFN‐γ showed the strongest association with t‐tau (*r* = 0.42, *p* < 0.001), followed by IL‐15 (*r* = 0.24, *p* < 0.001), TNF‐α (*r* = 0.23, *p* < 0.001), LIF (*r* = 0.22, *p* < 0.001), and IL‐18 (*r* = 0.20, *p* < 0.001). NfL correlated with IFN‐γ (*r* = 0.29, *p* < 0.001), CD30 (*r* = 0.26, *p* < 0.001), TNF‐RII (*r* = 0.21, *p* < 0.001), IL‐2R (*r* = 0.21, *p* < 0.001), and IL‐16 (*r* = 0.19, *p* < 0.001). GFAP correlated with IFN‐γ (*r* = 0.33, *p* < 0.001), LIF (*r* = 0.20, *p* < 0.001), and IL‐18 (*r* = 0.19, *p* < 0.001). Negative correlations were also identified between the Aβ42/40 ratio and IL‐17A (*r* = −0.18, *p* < 0.001) and IL‐7 (*r* = −0.19, *p* < 0.001).

In the AD group, negative correlations were observed between the Aβ42/40 ratio and MIF (*r* = −0.28, *p* < 0.001) and between Aβ40 and HGF (*r* = −0.25, *p* < 0.001). GFAP correlated with IL‐2R (*r* = 0.21, *p* < 0.001), and NfL correlated with SCF (*r* = 0.22, *p* < 0.001) and IL‐16 (*r* = 0.22, *p* < 0.001).

Across all groups, TNF‐RII and IL‐16 were the most consistent correlates of NfL and GFAP, while IL‐2R and SCF showed modest, stable associations across stages. No significant correlations (defined as *p* < 0.05, uncorrected) were detected between p‐tau217 and any of the inflammatory biomarkers.

### 3.5 Associations with clinical outcome measures

To investigate the clinical relevance of the inflammatory biomarkers, we examined the association of significant inflammatory markers with key clinical outcome scores using linear regression models adjusted for age, sex, years of education, and *APOE* ε4 carrier status. Initial analyses across the entire cohort identified numerous nominally significant associations (Table 3). To identify stage‐dependent relationships, these analyses were then stratified by cognitive group (CN, MCI, and mild AD), which showed distinct patterns of association (Table 4).

**TABLE 3 alz71257-tbl-0003:** Associations between plasma inflammatory markers and clinical outcome measures in total cohort (*N* = 954).

Clinical outcome	Biomarker	Coefficient (*β*)	Standard error (SE)	*p* value	Model adj. *R* ^2^
**MMSE (total score)**	APRIL	−0.4857	0.124	<0.001	0.117
	Eotaxin‐2 (CCL24)	−0.0635	0.03	0.037	0.107
	Eotaxin (CCL11)	−0.5381	0.137	<0.001	0.117
	GFAP	−0.004	0.001	<0.001	0.128
	HGF	−0.3969	0.152	0.009	0.109
	IFN‐γ	−0.2981	0.144	0.039	0.107
	IL‐15	−0.3788	0.106	<0.001	0.115
	IL‐16	−0.3406	0.154	0.028	0.107
	IL‐17A (CTLA‐8)	−0.1798	0.044	<0.001	0.118
	IL‐1β	−0.11	0.054	0.044	0.106
	IL‐7	−0.5311	0.09	<0.001	0.134
	LIF	−0.3651	0.082	<0.001	0.121
	MCP‐1 (CCL2)	−0.3891	0.119	0.001	0.113
	MDC	−0.0887	0.035	0.012	0.109
	MIP‐1β (CCL4)	−0.0984	0.044	0.026	0.107
	NfL	−0.0262	0.004	<0.001	0.148
	SCF	−0.2894	0.074	<0.001	0.117
	TNF‐α	−0.2678	0.07	<0.001	0.116
	TWEAK	−0.4021	0.177	0.024	0.107
**RAVLT (T‐score)**	APRIL	−1.2767	0.638	0.046	0.029
	Eotaxin‐2 (CCL24)	0.3491	0.155	0.024	0.03
	GFAP	−0.0113	0.004	0.004	0.033
	IL‐17A (CTLA‐8)	−1.2018	0.222	<0.001	0.054
	IL‐7	−1.854	0.465	<0.001	0.041
	NfL	−0.06	0.019	0.002	0.035
	SCF	−0.985	0.38	0.01	0.032
	TNF‐α	−1.1763	0.359	0.001	0.036
**FAQ (total score)**	APRIL	0.5407	0.248	0.03	0.073
	Eotaxin‐2 (CCL24)	0.1663	0.06	0.006	0.076
	Eotaxin (CCL11)	0.6362	0.273	0.02	0.074
	GFAP	0.0084	0.002	<0.001	0.098
	HGF	0.6633	0.302	0.028	0.073
	IL‐15	0.4998	0.212	0.018	0.074
	IL‐17A (CTLA‐8)	0.2004	0.088	0.022	0.073
	IL‐1β	0.2341	0.108	0.03	0.073
	IL‐7	0.6695	0.181	<0.001	0.082
	LIF	0.465	0.163	0.004	0.076
	MCP‐1 (CCL2)	0.5788	0.237	0.015	0.074
	MDC	0.1647	0.07	0.019	0.074
	NfL	0.0627	0.007	<0.001	0.137
	TNF‐α	0.3231	0.14	0.021	0.074
**GDS (total score)**	NfL	0.0059	0.003	0.021	0.018

*Note*: Results are derived from linear regression models adjusted for age, sex, years of education and *APOE* ε4 carrier status. The standardized regression coefficient (*β*) represents the effect size. A negative *β* indicates that higher biomarker levels are associated with poorer cognitive or functional outcomes, whereas a positive *β* indicates an association with betteroutcomes. Only nominally signifcant (p < 0.05, uncorrected) associations are displayed.

Abbreviations: AD, Alzheimer’s disease; CCL, C–C motif chemokine ligand; CN, cognitively normal; CTLA, cytotoxic T‐lymphocyte–associated protein; FAQ, Functional Activities Questionnaire; GDS, Geriatric Depression Scale; GFAP, glial fibrillary acidic protein; HGF, hepatocyte growth factor; IL, interleukin; MCI, mild cognitive impairment; MIP, macrophage inflammatory protein; MMSE, Mini‐Mental State Examination; NfL, neurofilament light chain; RAVLT, Rey Auditory Verbal Learning Test; SCF, stem cell factor; SE, standard error; TNF, tumor necrosis factor.

**TABLE 4 alz71257-tbl-0004:** Group‐specific associations between plasma inflammatory markers and clinical outcome measures.

Clinical outcome	Biomarker	Cognitive group	Coefficient (*β*)	Standard error (SE)	*p* value
**MMSE (total score)**	NfL	Mild AD	−0.017	0.005	0.002
	GFAP	Mild AD	−0.004	0.001	0.004
	IL‐17A (CTLA‐8)	Mild AD	−0.206	0.084	0.014
	IL‐7	Mild AD	−0.408	0.184	0.027
	IL‐18	MCI	−0.409	0.189	0.031
	APRIL	CN	−0.192	0.092	0.038
	MIP‐1β (CCL4)	CN	−0.072	0.032	0.025
	TNF‐α	CN	−0.137	0.057	0.016
**RAVLT (T‐score)**	Eotaxin‐2 (CCL24)	CN	0.463	0.202	0.022
		MCI	1.016	0.234	<0.001
		Mild AD	1.168	0.304	<0.001
	IL‐2R	MCI	2.353	0.826	0.005
		Mild AD	2.142	1.002	0.034
	IL‐17A (CTLA‐8)	CN	−1.203	0.327	<0.001
		MCI	−0.761	0.309	0.014
		Mild AD	−0.886	0.401	0.028
**FAQ (total score)**	SCF	CN	0.142	0.064	0.028
		MCI	−0.52	0.213	0.015
	MIP‐1α (CCL3)	Mild AD	−0.914	0.352	0.010
	NfL	MCI	0.023	0.009	0.013*
		Mild AD	0.058	0.014	<0.001
**GDS (total score)**	HGF	MCI	0.45	0.223	0.044

*Note*: Results are derived from linear regression models adjusted for age, sex, years of education and *APOE* ε4 carrier status. The standardized regression coefficient (*β*) represents the effect size. A negative *β* indicates that higher biomarker levels are associated with poorer cognitive or functional outcomes, whereas a positive *β* indicates an association with betteroutcomes. Only nominally signifcant (p < 0.05, uncorrected) associations are displayed.

Associations are presented separately for CN, MCI and mild AD groups. Results are from linear regression models adjusted for age, sex, years of education and *APOE* ε4 carrier status. Only nominally significant (*p* < 0.05, uncorrected) associations are displayed.

Abbreviations: AD, Alzheimer's disease; *β*, standardized regression coefficient; CCL, C–C motif chemokine ligand; CN, cognitively normal; CTLA, cytotoxic T‐lymphocyte–associated protein; FAQ, Functional Activities Questionnaire; GDS, Geriatric Depression Scale; GFAP, glial fibrillary acidic protein; HGF, hepatocyte growth factor; IL, interleukin; MCI, mild cognitive impairment; MIP, macrophage inflammatory protein; MMSE, Mini‐Mental State Examination; NfL, neurofilament light chain; RAVLT, Rey Auditory Verbal Learning Test; SE, standard error; SCF, stem cell factor; TNF, tumor necrosis factor.

#### 3.5.1 Global cognition (MMSE)

Inflammatory biomarker associations with MMSE were found to be dependent on cognitive stage. In the mild AD group, higher plasma levels of NfL (*β* = −0.017, SE = 0.005, *p* = 0.002), GFAP (*β* = −0.004, SE = 0.001, *p* = 0.004), IL‐17A (CTLA‐8) (*β* = −0.206, SE = 0.084, *p* = 0.014), and IL‐7 (*β* = −0.408, SE = 0.184, *p* = 0.027) were all linked to lower MMSE scores,[Table alz71257-tbl-0003], [Table alz71257-tbl-0004] associations that were not statistically significant in the CN or MCI groups. In the MCI group, only higher IL‐18 was associated with poorer MMSE scores, with a notable effect size (*β* = −0.409, SE = 0.189, *p* = 0.031). Interestingly, nominally significant associations were also found in the CN group, suggesting a link between inflammation and subtle cognitive performance even in healthy individuals. Associations found in the CN group had more modest effect sizes, where higher levels of APRIL (*β* = −0.192, SE = 0.092, *p* = 0.038), MIP‐1β (CCL4) (*β* = −0.072, SE = 0.032, *p* = 0.025), and TNF‐α (*β* = −0.137, SE = 0.057, *p* = 0.016) were associated with lower MMSE scores.

#### 3.5.2 Memory performance (RAVLT delayed recall)

Associations with RAVLT T scores (memory performance) were also specific to cognitive stage. Higher levels of IL‐17A (CTLA‐8) were associated with worse memory performance across all three groups, where magnitude of the association was strongest in the CN group (*β* = −1.203, SE = 0.327, *p* < 0.001) and remained significant but less pronounced in the MCI (*β* = −0.761, SE = 0.309, *p* = 0.014) and mild AD (*β* = −0.886, SE = 0.401, *p* = 0.028) groups. Higher levels of IL‐7 and MIP‐1β (CCL4) were associated with poorer memory specifically in the CN group.

Eotaxin‐2 (CCL24) was linked to better RAVLT scores in all three groups, with a graded effect as the strength of this association increased from CN (*β* = 0.463) to MCI (*β* = 1.016) and was strongest in the mild AD group (*β* = 1.168). Similarly, higher IL‐2R was associated with better memory in both MCI (*β* = 2.353, SE = 0.826, *p* = 0.005) and mild AD (*β* = 2.142, SE = 1.002, *p* = 0.034) groups, but not in the CN group.

The mild AD group showed a number of associations with better RAVLT scores, where higher levels of eotaxin (CCL11), IFN‐γ, IL‐16, IL‐1β, IP‐10 (CXCL10), MIF, MIP‐1α (CCL3), MIP‐1β (CCL4), and TWEAK were all linked to better memory.

#### 3.5.3 Functional ability (FAQ)

Associations with functional ability (FAQ), where higher scores indicate greater functional impairment, were also dependent on cognitive stage. There was a link between neurodegeneration and functional decline in the symptomatic stages, with higher NfL levels associated with worse function in both MCI (*β* = 0.023, SE = 0.009, *p* = 0.013) and mild AD (*β* = 0.058, SE = 0.014, *p* < 0.001) groups, an association not present in the CN group.

A different pattern emerged in the CN group, where a distinct set of markers, including eotaxin‐2 (CCL24), IL‐16, IL‐2R, and MCP‐1 (CCL2), were uniquely associated with worse function. An interesting pattern was observed for SCF, with higher levels associated with worse function (*β* = 0.142, SE = 0.064, *p* = 0.028) in the CN group, whereas in the MCI group, higher levels were associated with better function (*β* = −0.520, SE = 0.213, *p* = 0.015), suggesting a possible shift in its role during disease progression.

#### 3.5.4 Depressive symptoms (GDS)

Associations with depressive symptoms (GDS) were few, and the main finding was of higher HGF levels associated with worse mood in the MCI group, with a notable effect size (*β* = 0.450, SE = 0.223, *p* = 0.044). No other biomarkers showed a statistically significant association with GDS scores in any of the cognitive groups.

## 4 DISCUSSION

### 4.1 Summary of findings

Our study provides a comprehensive analysis of plasma inflammatory biomarker profiles across the AD spectrum within the Bio‐Hermes cohort. These revealed stage‐dependent patterns, including both amyloid‐dependent and amyloid‐independent pathways, and some biomarkers showed paradoxical associations with clinical outcomes, suggesting inflammation may not be uniformly detrimental and could have context‐dependent roles.

### 4.2 Amyloid‐dependent and amyloid‐independent inflammatory profiles

Our amyloid‐stratified approach revealed a core signature of 15 markers (14 cytokines and NfL) associated with cognitive decline regardless of amyloid status, suggesting amyloid‐independent processes relating to other drivers of neurodegeneration, such as cerebrovascular disease or primary age‐related tauopathy.^17^ In contrast, amyloid‐positive individuals displayed a broader, distinct inflammatory profile, characterized by GFAP and seven cytokines (IL‐1β, IL‐18, IL‐12p70, CCL2, CCL3, IL‐15, and TNF‐RII) significantly elevated only in this subgroup. Among these, IL‐1β and IL‐18, products of the canonical NLRP3 inflammasome, implicate microglial reactivity as a key amyloid‐related process. These were elevated in mild AD, and higher IL‐18 associated with poorer cognition in MCI, consistent with prior evidence that amyloid‐related inflammasome activation links to neuronal injury.^8^ GFAP elevation reflects reactive astrocytosis, while increased CCL2 and CCL3, chemokines released by activated astrocytes and microglia, suggest recruitment of peripheral monocytes and amplification of local neuroinflammation. TNF‐RII and IL‐12p70 levels indicate sustained TNF and T helper (Th) 1‐type immune activation, reflecting chronic inflammatory signaling, while IL‐15, which supports Natural Killer‐cell and T‐cell survival, suggests ongoing peripheral immune engagement that may reinforce central pathology. Collectively, these findings support a model where amyloid pathology triggers an amyloid‐driven glial–immune cascade, linking microglial inflammasome activation, astrocytic reactivity, and peripheral immune recruitment. This profile is consistent with a “canonical AD inflammatory” subtype and extends existing models of amyloid‐related microglial activation by revealing coordinated astrocytic and systemic immune components. Consistent with this interpretation, preclinical studies show that pharmacological inhibition of NLRP3 can attenuate both amyloid and tau pathology, supporting the hypothesis that inflammasome‐mediated glial–immune activation represents a mechanistic and potentially targetable pathway within this canonical AD inflammatory subtype.^8^, ^9^

By comparison, the amyloid‐negative subgroup exhibited a more restricted inflammatory profile, characterized solely by elevated IL‐2R, suggesting a distinct, potentially compensatory or reparative immune response. Elevated IL‐2R could indicate enhanced T‐regulatory activity, consistent with an anti‐inflammatory or restorative mechanism that may attempt to counteract neurodegenerative processes. This finding aligns with emerging evidence that regulatory T‐cell signaling may influence neuroinflammation and neuroprotection in non‐amyloid pathologies,^18^ including cerebrovascular and primary tau‐related disease.^19^ This raises the possibility that immune‐modulating therapies targeting T‐cell pathways, such as low‐dose IL‐2 therapy, could be of benefit in this subgroup.

### 4.3 Stage‐dependent mechanisms and compensatory signatures

Stage‐specific analyses suggest that inflammation may act as a driver, concurrent process, or consequence of neurodegeneration, depending on disease stage. In the CN group, higher APRIL, MIP‐1β (CCL4), and TNF‐α were associated with lower MMSE scores, suggesting subtle systemic immune changes may precede clinical symptoms. In MCI, IFN‐γ strongly correlated with t‐tau, and higher IL‐18 was associated with lower MMSE scores, implicating inflammation as a concurrent process in the disease transition. By contrast, in mild AD, the broad elevation of 18 cytokines, together with correlations between multiple cytokines (including IL‐17A, IL‐7, GFAP, and NfL) and lower MMSE scores, indicates that inflammation becomes increasingly consequential to neurodegeneration.^20^

Paradoxical associations were also identified, consistent with work reporting that immune protein may support cognitive resilience despite pathology, challenging the “more inflammation is worse” model.^21^ These included eotaxin‐2 (CCL24), consistently linked to better RAVLT performance across all groups, with a graded effect from CN through MCI to mild AD. Similarly, IL‐2R predicted better memory in MCI and AD. In mild AD, several additional cytokines, including eotaxin, IFN‐γ, IL‐16, IL‐1β, IP‐10, MIF, and TWEAK, were positively associated with memory, suggesting compensatory immune mechanisms at later stages. Functional outcomes showed similar stage‐dependent profiles: Higher NfL predicted worse function in MCI and mild AD, whereas in CN, cytokines such as eotaxin‐2, IL‐16, and IL‐2R were associated with poorer function, and SCF showed opposite effects across stages.

Beyond individual cytokines, we observed coordinated correlation‐based serum immune signatures, including a large inflammatory hub centered on TNF‐RII and its co‐regulated partners (IL‐16, MIF, HGF, TWEAK, Eotaxin, APRIL, and VEGF‐A), alongside an IL‐17A–IL‐7 axis and an IFN‐γ–IL‐18 hub, indicating that inflammatory signals in AD tend to vary in correlated sets. These are consistent with the proposed amyloid‐driven glial–immune cascade, reflecting synchronized activation across microglial, astrocytic, and peripheral immune pathways.^8^, ^22^ Similar network‐level co‐activation of glial and immune pathways have also been reported in AD brain tissue, supporting the biological plausibility of these coordinated patterns.^22^ Consistent with prior findings, NfL correlated positively with TNF‐RII across all clinical stages, reinforcing a link between peripheral TNF‐pathway activity and neuroaxonal injury.

Our data also extend previous reports of IL‐17A‐related mechanisms, showing that higher IL‐17A and IL‐7 levels were associated with a more pathological Aβ42/40 ratio in MCI, suggesting early Th17‐type involvement. While the exact composition of the identified correlation‐based groupings (plasma “hubs”) appear novel, they are relatable to pathway‐level coordination across datasets, including transcriptomic and proteomic studies of AD brain and plasma.^23^, ^24^ VEGF‐A's correlations with APRIL and other hub cytokines further suggest links between angiogenesis and immune regulation.

### 4.4 Genetic and sustained inflammatory pathways

Genetic risk further shaped inflammatory responses. *APOE* ε4, was associated with higher GFAP, consistent with reports of ε4‐linked astroglial reactivity and heightened vulnerability to amyloid pathology^25^, ^26^ We also observed lower circulating IL‐15, IL‐20, and MCP‐2/CCL8 in carriers compared to non‐carriers, despite higher IL‐15 and MCP‐2 in mild AD overall. This pattern suggests ε4 status may shape a baseline immune profile distinct from disease‐stage effects.

TNF‐RII hub members showed consistent positive correlations with markers of neurodegeneration (NfL, GFAP) across all disease stages, indicating that TNF‐pathway activation represents a sustained rather than progressively amplifying inflammatory process. This stability supports a model in which chronic TNF‐mediated signaling contributes to ongoing glial and peripheral immune activation, providing a rationale for evaluating selective TNF‐α inhibitors in later‐stage disease, as suggested by epidemiological studies.^7^, ^20^

Together, these insights extend beyond biomarker identification, offering a framework for mechanism‐based stratification of patients. Longitudinal studies and replication in diverse cohorts will be critical to validate these mechanistic subtypes and to guide precision medicine through targeted clinical trials.

### 4.5 Limitations and future directions

This study has some limitations. First, its cross‐sectional design precludes causal inference regarding the directionality of the amyloid–immune–cognition axis. The associations reported are hypothesis generating signals rather than mechanistic pathways. Longitudinal studies are required to establish temporal relationships and clarify whether inflammatory changes precede, accompany, or follow amyloid pathology and neurodegeneration. Second, plasma cytokine measurements may not fully reflect central neuroinflammatory processes. Although the cytokines included cross or affect the blood—brain barrier (BBB), peripheral levels may not accurately capture brain‐specific immune responses. This challenge is complicated by BBB dysfunction in AD, which could distort peripheral measurements and contribute to systemic–central inflammatory feedback. Future work integrating CSF and plasma cytokine data will be essential.

Third, while multiplex panels allow for broad profiling, they are limited by variable sensitivity and detection thresholds. In our study, 33 cytokines were excluded because over half of the values fell below the reportable range. Although this reduced noise, it restricted evaluation of several cytokines implicated in AD pathophysiology (e.g., IL‐4, IL‐10, TGF‐β) and limited interpretation of how excluded markers might interact with the inflammatory signatures we identified. Future studies should use higher‐sensitivity assays to detect low‐abundance cytokines. Fourth, the absence of clinically established cut‐offs for cytokines hinders direct translation. This reflects a limitation in the field, where inflammatory markers remain largely exploratory and lack validated thresholds. The standardization of cytokine assays and development of reference ranges will be crucial for clinical application.

Finally, although we adjusted for key biological covariates (age, sex, *APOE* ε4), we did not include specific comorbidities or medication classes in our models, which can modulate systemic inflammation. The Bio‐Hermes dataset captures these data in extensive detail, containing a large number of medication names and comorbid conditions, including these directly as multiple covariates would introduce excessive dimensionality and risk model instability. Moreover, our aim was to characterize the total inflammatory profile of the clinical AD population, where multimorbidity is common. Future studies should address these factors more systematically by calculating validated composite measures, such as the Charlson Comorbidity Index, to disentangle the effects of multimorbidity and medication use from AD‐specific inflammatory signaling.

Our study provides initial evidence that plasma immune signatures can differentiate clinical stage, amyloid status, and genetic risk in a deeply phenotyped, large cohort. The identification of amyloid‐dependent versus independent inflammatory pathways, including distinct glial and peripheral immune signatures, supports an integrative framework for understanding immune heterogeneity in AD. Future work should focus on (1) validating these cytokine signatures in longitudinal cohorts, (2) integrating plasma, CSF, and imaging measures, (3) establishing the clinical utility of composite inflammatory scores for prognosis, and (4) testing pathway‐specific immunomodulators in stratified clinical trials (particularly targeting inflammasome and TNF pathways, given the failure of previous trials using nonsteroidal anti‐inflammatory drugs, which may have inadvertently inhibited potentially beneficial or context‐dependent immune responses^27^).

## 5 CONCLUSION

In conclusion, this exploratory study demonstrates that systemic inflammation is integral to AD pathophysiology. Amyloid‐stratified analyses revealed a core amyloid‐independent inflammatory signature and a broader amyloid‐specific profile involving coordinated glial and peripheral immune activation, suggesting distinct biological pathways to neurodegeneration. Stage‐dependent associations indicate that inflammatory processes may act as early drivers, concurrent mechanisms, or late amplifiers of injury. Collectively, these findings highlight inflammatory heterogeneity as a key contributor to AD and support a hypothesis‐generating framework to guide validation in longitudinal cohorts and inform the design of targeted, stage‐specific immunomodulatory trials.

## CONFLICT OF INTEREST STATEMENT

CWR is CEO, founder, and majority shareholder in Scottish Brain Sciences, which has or held contracts with Lilly, Merck, Linus Health, Janssen Cilag, Roche, Roche Diagnostics, Thereni, Biogen, and Eisai. ZH, DN, SL, FD, and JC have no disclosures.

## CONSENT STATEMENT

All Bio‐Hermes participants provided written informed consent under central International Review Board approval (Advarra; Protocol Pro00046018). Our secondary analysis used de‐identified data under a data‐use agreement, and no additional participant consent was required.

## Supporting information



Supporting information

Supporting information

Supporting information

Supporting information
