# Supplementary material for: Plasma inflammatory biomarker profiles across the Alzheimer's disease spectrum in the Bio‐Hermes cohort
Source: Alzheimers Dement. 2026 Mar 12;22(3):e71257. doi: 10.1002/alz.71257 (PMC13093584; doi:10.1002/alz.71257)
Supplement: Supplementary file 1 — Supporting information [file ALZ-22-e71257-s002.docx]

**Supplementary Table S1. Proportion of undetectable plasma cytokine values**

**Table S1.** Proportion of undetectable (OOR<) values for all plasma cytokines measured in the Bio-Hermes cohort. Cytokines with >50% undetectable values were excluded from downstream analyses.

| **Cytokine** | **% Undetectable (OOR<)** | **Included in analysis** |
| --- | --- | --- |
| APRIL | 0.40 | Yes |
| BAFF | 96.82 | No (>50%) |
| BLC (CXCL13) | 91.54 | No (>50%) |
| CD30 | 0.00 | Yes |
| CD40L | 56.82 | No (>50%) |
| ENA-78 (CXCL5) | 0.00 | Yes |
| Eotaxin (CCL11) | 0.10 | Yes |
| Eotaxin-2 (CCL24) | 13.73 | Yes |
| Eotaxin-3 (CCL26) | 95.82 | No (>50%) |
| FGF-2 | 88.46 | No (>50%) |
| Fractalkine (CX3CL1) | 74.13 | No (>50%) |
| G-CSF (CSF-3) | 95.62 | No (>50%) |
| GM-CSF | 59.50 | No (>50%) |
| GRO alpha (CXCL1) | 89.65 | No (>50%) |
| HGF | 0.00 | Yes |
| I-TAC (CXCL11) | 51.04 | No (>50%) |
| IFN alpha | 96.72 | No (>50%) |
| IFN gamma | 0.80 | Yes |
| IL-1 alpha | 96.92 | No (>50%) |
| IL-1 beta | 28.06 | Yes |
| IL-2 | 94.03 | No (>50%) |
| IL-2R | 1.00 | Yes |
| IL-3 | 81.29 | No (>50%) |
| IL-4 | 86.17 | No (>50%) |
| IL-5 | 83.68 | No (>50%) |
| IL-6 | 87.66 | No (>50%) |
| IL-7 | 5.77 | Yes |
| IL-8 (CXCL8) | 86.87 | No (>50%) |
| IL-9 | 82.29 | No (>50%) |
| IL-10 | 84.28 | No (>50%) |
| IL-12p70 | 10.85 | Yes |
| IL-13 | 78.91 | No (>50%) |
| IL-15 | 7.06 | Yes |
| IL-16 | 0.00 | Yes |
| IL-17A (CTLA-8) | 10.45 | Yes |
| IL-18 | 0.40 | Yes |
| IL-20 | 33.43 | Yes |
| IL-21 | 76.92 | No (>50%) |
| IL-22 | 48.66 | Yes |
| IL-23 | 88.66 | No (>50%) |
| IL-27 | 92.34 | No (>50%) |
| MCP-1 (CCL2) | 0.20 | Yes |
| MCP-2 (CCL8) | 0.20 | Yes |
| MCP-3 (CCL7) | 86.17 | No (>50%) |
| MDC | 16.12 | Yes |
| MIF | 0.00 | Yes |
| MIG (CXCL9) | 81.59 | No (>50%) |
| MIP-1 alpha (CCL3) | 7.86 | Yes |
| MIP-1 beta (CCL4) | 7.96 | Yes |
| MIP-3 alpha (CCL20) | 46.97 | Yes |
| MMP-1 | 64.78 | No (>50%) |
| NGF beta | 83.48 | No (>50%) |
| SCF | 9.35 | Yes |
| SDF-1 alpha | 66.57 | No (>50%) |
| TNF alpha | 6.77 | Yes |
| TNF beta | 92.44 | No (>50%) |
| TNF-RII | 0.00 | Yes |
| TRAIL | 53.63 | No (>50%) |
| TSLP | 26.37 | Yes |
| TWEAK | 0.00 | Yes |
| VEGF-A | 2.09 | Yes |
