# Supplementary material for: Plasma inflammatory biomarker profiles across the Alzheimer's disease spectrum in the Bio‐Hermes cohort
Source: Alzheimers Dement. 2026 Mar 12;22(3):e71257. doi: 10.1002/alz.71257 (PMC13093584; doi:10.1002/alz.71257)
Supplement: Supplementary file 2 — Supporting information [file ALZ-22-e71257-s001.docx]

**Supplementary Table S2. Comprehensive Partial Correlations between Plasma Cytokines and AD Biomarkers**

**Table S3.** Partial Pearson correlations (r) between plasma cytokines (log-transformed) and core AD biomarkers (Aβ40, Aβ42, Aβ42/40, p-tau181, p-tau217, t-tau, NfL, GFAP), adjusted for age, sex, and APOE ε4 carrier status. Only nominally significant correlations (p < 0.05, uncorrected) are displayed. Cells marked with "—" indicate non-significant relationships (p ≥ 0.05).

**Table S3A. Cognitively Normal (CN) Group (n=404)**

| **Cytokine** | **Aβ40 (r)** | **Aβ42 (r)** | **Aβ42/40 (r)** | **p-tau181 (r)** | **p-tau217 (r)** | **t-tau (r)** | **NfL (r)** | **GFAP (r)** |
| --- | --- | --- | --- | --- | --- | --- | --- | --- |
| **APRIL** | — | -0.137 | — | — | — | — | 0.157 | 0.153 |
| **CD30** | 0.107 | — | — | — | — | — | 0.241 | 0.163 |
| **ENA-78 (CXCL5)** | -0.208 | -0.197 | — | — | — | — | — | — |
| **Eotaxin (CCL11)** | — | -0.126 | — | — | — | — | 0.193 | 0.137 |
| **Eotaxin-2 (CCL24)** | — | — | — | — | — | — | 0.122 | — |
| **HGF** | — | — | -0.121 | — | — | — | 0.106 | — |
| **IFN-γ** | — | — | — | — | — | — | — | — |
| **IL-1β** | — | — | — | — | -0.106 | — | — | — |
| **IL-2R** | 0.106 | — | — | — | 0.108 | — | 0.230 | 0.158 |
| **IL-7** | -0.190 | -0.225 | — | — | — | — | — | 0.104 |
| **IL-12p70** | — | — | -0.103 | — | — | — | — | — |
| **IL-15** | — | — | — | — | — | — | — | — |
| **IL-16** | — | — | — | — | — | 0.167 | 0.234 | 0.100 |
| **IL-17A (CTLA-8)** | -0.182 | -0.177 | — | — | — | — | — | — |
| **IL-18** | — | — | — | — | — | — | — | 0.145 |
| **IL-20** | — | — | — | — | — | — | — | 0.122 |
| **IL-22** | — | — | — | — | — | — | — | — |
| **IP-10 (CXCL10)** | — | — | — | — | — | — | 0.143 | — |
| **LIF** | — | — | — | — | — | — | — | — |
| **MCP-1 (CCL2)** | — | — | — | — | — | — | 0.124 | — |
| **MCP-2 (CCL8)** | 0.150 | — | -0.114 | — | — | 0.113 | — | — |
| **MDC** | 0.107 | — | -0.107 | — | — | — | — | — |
| **MIF** | — | — | — | — | — | 0.121 | 0.173 | — |
| **MIP-1α (CCL3)** | — | — | — | — | — | — | — | — |
| **MIP-1β (CCL4)** | — | — | — | — | — | — | 0.109 | 0.114 |
| **MIP-3α (CCL20)** | — | -0.144 | — | — | — | — | — | — |
| **SCF** | — | — | — | 0.100 | — | 0.106 | 0.178 | 0.176 |
| **TNF-α** | -0.188 | -0.186 | — | — | — | — | — | — |
| **TNF-RII** | — | — | — | — | — | 0.148 | 0.272 | 0.144 |
| **TSLP** | — | — | — | — | -0.099 | — | — | — |
| **TWEAK** | — | -0.102 | — | — | — | — | 0.156 | 0.154 |
| **VEGF-A** | — | — | — | — | — | — | 0.116 | — |

**Table S3B. Mild Cognitive Impairment (MCI) Group (n=302)**

| **Cytokine** | **Aβ40 (r)** | **Aβ42 (r)** | **Aβ42/40 (r)** | **p-tau181 (r)** | **p-tau217 (r)** | **t-tau (r)** | **NfL (r)** | **GFAP (r)** |
| --- | --- | --- | --- | --- | --- | --- | --- | --- |
| **APRIL** | — | — | — | — | 0.157 | 0.127 | 0.145 | — |
| **CD30** | 0.199 | 0.150 | — | — | — | 0.144 | 0.256 | 0.180 |
| **Eotaxin (CCL11)** | — | — | — | — | — | 0.144 | 0.156 | 0.123 |
| **IFN-γ** | — | 0.123 | — | — | — | 0.416 | 0.294 | 0.326 |
| **IL-1β** | — | — | — | — | — | 0.168 | 0.139 | 0.165 |
| **IL-2R** | 0.165 | — | — | — | — | — | 0.211 | — |
| **IL-12p70** | 0.114 | — | — | — | — | 0.185 | 0.160 | 0.149 |
| **IL-15** | — | — | — | — | — | 0.244 | 0.166 | 0.161 |
| **IL-16** | — | — | — | 0.133 | — | — | 0.194 | — |
| **IL-18** | — | — | — | — | 0.114 | 0.203 | 0.200 | 0.195 |
| **IL-22** | — | — | — | — | 0.153 | 0.133 | 0.128 | 0.135 |
| **LIF** | — | — | — | — | — | 0.220 | 0.171 | 0.199 |
| **MCP-1 (CCL2)** | — | — | — | — | — | 0.117 | — | — |
| **MCP-2 (CCL8)** | 0.131 | — | — | — | — | 0.190 | 0.126 | — |
| **MDC** | 0.128 | — | — | — | — | — | — | — |
| **MIF** | — | — | — | — | — | — | 0.127 | — |
| **MIP-3α (CCL20)** | — | — | — | — | — | 0.168 | 0.139 | — |
| **SCF** | 0.143 | — | — | — | — | — | 0.119 | — |
| **TNF-α** | — | — | — | — | — | 0.225 | — | 0.149 |
| **TNF-RII** | 0.155 | — | — | — | 0.174 | — | 0.211 | — |
| **TSLP** | — | — | — | — | — | 0.145 | — | — |
| **VEGF-A** | 0.127 | — | — | — | 0.168 | 0.117 | 0.134 | — |

**Table S3C. Mild Alzheimer’s Disease (Mild AD) Group (n=258)**

| **Cytokine** | **Aβ40 (r)** | **Aβ42 (r)** | **Aβ42/40 (r)** | **p-tau181 (r)** | **p-tau217 (r)** | **t-tau (r)** | **NfL (r)** | **GFAP (r)** |
| --- | --- | --- | --- | --- | --- | --- | --- | --- |
| **APRIL** | — | — | — | — | — | — | 0.153 | — |
| **CD30** | — | — | — | -0.135 | — | — | 0.196 | — |
| **ENA-78 (CXCL5)** | — | — | — | — | — | -0.137 | — | — |
| **Eotaxin (CCL11)** | -0.257 | -0.294 | 0.166 | — | — | — | 0.166 | 0.134 |
| **Eotaxin-2 (CCL24)** | -0.184 | -0.188 | — | — | — | — | 0.158 | — |
| **HGF** | -0.224 | -0.213 | 0.248 | — | — | — | 0.169 | — |
| **IL-1β** | — | — | — | — | — | — | 0.156 | — |
| **IL-2R** | — | — | 0.188 | — | 0.167 | — | 0.149 | 0.213 |
| **IL-7** | — | -0.144 | — | — | — | — | — | — |
| **IL-12p70** | — | — | — | — | — | — | 0.135 | — |
| **IL-15** | — | — | — | — | — | — | 0.192 | 0.169 |
| **IL-16** | -0.131 | -0.168 | 0.156 | — | — | 0.154 | 0.221 | 0.131 |
| **IL-20** | — | -0.201 | — | — | — | — | — | — |
| **IL-22** | — | — | — | — | — | 0.124 | — | — |
| **IP-10 (CXCL10)** | -0.344 | -0.367 | 0.187 | -0.179 | — | — | — | — |
| **LIF** | — | — | — | — | — | — | 0.133 | — |
| **MCP-1 (CCL2)** | — | — | 0.134 | — | — | — | 0.173 | 0.154 |
| **MCP-2 (CCL8)** | — | — | 0.162 | — | — | — | — | — |
| **MIF** | -0.278 | -0.254 | 0.278 | — | — | — | — | — |
| **MIP-1α (CCL3)** | — | -0.153 | — | — | — | — | — | — |
| **MIP-1β (CCL4)** | -0.127 | -0.142 | — | — | — | — | 0.142 | — |
| **MIP-3α (CCL20)** | — | — | — | — | — | — | 0.127 | — |
| **SCF** | — | — | — | — | — | — | 0.221 | 0.179 |
| **TNF-α** | — | — | — | 0.132 | — | — | — | — |
| **TNF-RII** | -0.220 | -0.255 | 0.195 | — | — | — | 0.193 | 0.129 |
| **TWEAK** | -0.225 | -0.249 | — | — | — | — | — | — |
| **VEGF-A** | — | — | — | — | — | — | 0.200 | — |
