# Supplementary material for: Plasma inflammatory biomarker profiles across the Alzheimer's disease spectrum in the Bio‐Hermes cohort
Source: Alzheimers Dement. 2026 Mar 12;22(3):e71257. doi: 10.1002/alz.71257 (PMC13093584; doi:10.1002/alz.71257)
Supplement: Supplementary file 3 — Supporting information [file ALZ-22-e71257-s003.docx]

## Supplementary Table S3. Amyloid-stratified differences in inflammatory biomarkers across cognitive groups

Pairwise cognitive group comparisons (CN, MCI, AD) following ANCOVA adjusted for age and sex, stratified by amyloid status. Shown are post-hoc Tukey contrasts with effect sizes (Cohen’s *d*); *q*-values are FDR-corrected within each amyloid stratum.

## Supplementary Table S3A. Amyloid-negative participants (Aβ−)

| Inflammatory biomarker | Cognitive group comparison | Mean difference | Cohen’s d | q value (FDR) |
| --- | --- | --- | --- | --- |
| APRIL | CN vs AD | 0.303 | −0.42 | 0.003 |
| Eotaxin-2 (CCL24) | CN vs MCI | 0.845 | −0.27 | 0.026 |
| Eotaxin-2 (CCL24) | CN vs AD | 1.519 | −0.47 | <0.001 |
| Eotaxin (CCL11) | CN vs AD | 0.335 | −0.49 | <0.001 |
| HGF | CN vs MCI | 0.168 | −0.27 | 0.026 |
| HGF | CN vs AD | 0.310 | −0.48 | <0.001 |
| IL-16 | CN vs AD | 0.225 | −0.37 | 0.011 |
| IL-2R | CN vs MCI | 0.266 | −0.31 | 0.010 |
| IL-7 | CN vs AD | 0.450 | −0.45 | 0.002 |
| IL-7 | MCI vs AD | 0.418 | −0.43 | 0.010 |
| IP-10 (CXCL10) | CN vs AD | 0.268 | −0.42 | 0.004 |
| LIF | CN vs AD | 0.578 | −0.54 | <0.001 |
| MCP-2 (CCL8) | CN vs MCI | 0.167 | −0.27 | 0.025 |
| MCP-2 (CCL8) | CN vs AD | 0.193 | −0.30 | 0.039 |
| MDC | CN vs AD | 1.053 | −0.40 | 0.008 |
| MDC | MCI vs AD | 0.905 | −0.37 | 0.039 |
| MIF | CN vs AD | 0.286 | −0.51 | <0.001 |
| MIF | MCI vs AD | 0.200 | −0.33 | 0.029 |
| MIP-1β (CCL4) | CN vs AD | 0.761 | −0.38 | 0.011 |
| NfL | CN vs MCI | 9.462 | −0.41 | <0.001 |
| NfL | CN vs AD | 12.240 | −0.80 | <0.001 |
| SCF | CN vs AD | 0.559 | −0.46 | 0.002 |
| SCF | MCI vs AD | 0.402 | −0.34 | 0.049 |
| TWEAK | CN vs AD | 0.170 | −0.33 | 0.036 |

## Supplementary Table S3B. Amyloid-positive participants (Aβ+)

| Inflammatory biomarker | Cognitive comparison | Mean difference | Cohen’s d | q value (FDR) |
| --- | --- | --- | --- | --- |
| APRIL | MCI vs AD | 0.310 | −0.46 | 0.019 |
| Eotaxin-2 (CCL24) | CN vs AD | 1.400 | −0.52 | 0.003 |
| Eotaxin (CCL11) | CN vs AD | 0.426 | −0.66 | <0.001 |
| Eotaxin (CCL11) | MCI vs AD | 0.406 | −0.70 | <0.001 |
| GFAP | CN vs AD | 61.507 | −0.54 | 0.002 |
| GFAP | MCI vs AD | 41.287 | −0.35 | 0.034 |
| HGF | CN vs AD | 0.316 | −0.58 | 0.002 |
| HGF | MCI vs AD | 0.223 | −0.41 | 0.021 |
| IL-15 | CN vs AD | 0.554 | −0.65 | <0.001 |
| IL-15 | MCI vs AD | 0.431 | −0.55 | 0.002 |
| IL-16 | CN vs AD | 0.247 | −0.42 | 0.019 |
| IL-16 | MCI vs AD | 0.243 | −0.45 | 0.015 |
| IL-7 | CN vs AD | 0.379 | −0.41 | 0.024 |
| IL-7 | MCI vs AD | 0.532 | −0.59 | <0.001 |
| IP-10 (CXCL10) | CN vs AD | 0.341 | −0.50 | 0.003 |
| LIF | CN vs AD | 0.486 | −0.44 | 0.015 |
| MCP-1 (CCL2) | CN vs AD | 0.308 | −0.41 | 0.021 |
| MCP-1 (CCL2) | MCI vs AD | 0.352 | −0.48 | 0.004 |
| MDC | CN vs AD | 1.203 | −0.57 | 0.003 |
| MDC | MCI vs AD | 1.180 | −0.54 | 0.002 |
| MIF | CN vs AD | 0.212 | −0.40 | 0.023 |
| MIP-1α (CCL3) | CN vs AD | 0.510 | −0.42 | 0.022 |
| MIP-1β (CCL4) | CN vs AD | 0.898 | −0.44 | 0.019 |
| NfL | CN vs AD | 16.493 | −0.60 | <0.001 |
| NfL | MCI vs AD | 12.758 | −0.45 | 0.003 |
| SCF | MCI vs AD | 0.588 | −0.52 | 0.002 |
| TNF-RII | CN vs AD | 0.226 | −0.44 | 0.019 |
| TNF-RII | MCI vs AD | 0.182 | −0.36 | 0.049 |
| TWEAK | CN vs AD | 0.202 | −0.41 | 0.019 |
| TWEAK | MCI vs AD | 0.277 | −0.63 | <0.001 |
